# The Street Walker

**Published:** 1916-04

**Authors:** 


### The Street Walker

We resolved, on entering upon the editorial management of the BUFFALO MEDICAL JOURNAL, to discuss sexual problems as little as possible. Unfortunately, such problems always are of vital interest and there seems to be an unprecedented amount of attention paid to them at present by lay societies and periodicals. Hence, we can not well avoid mentioning them.

In a recent visit to New York, we attended a session of the women's night court, which seems to specialize in street walking cases. Special detectives, young men in plain clothes, appeared in succession with their catches, gave the same testimony of being on such and such a street, seeing the girl walk so many feet north or south, accost one man after another and then of making the arrest. The girls usually waived subsequent trial with an attorney, sometimes made hysteric denials, sometimes an admission, sometimes lied about previous convictions, were sentenced to penal or reformatory institutions. The matron, a sweet-faced, white-haired woman, stated that reform was seldom accomplished, that the work was mainly one of "street cleaning." From other sources, accounts were received of painful errors in the way of arrests of perfectly innocent women and girls, talking with friends and relatives, or asking directions from strangers. On the streets, in the evenings, we could see no obvious diminution of the trade by this system of street cleaning, in fact we were accosted while waiting in front of a store within which most of our near female relatives were making purchases.

That the street walker is an evil, that she is a very direct etiologic factor in regard to disease, goes without saying. But she is not primarily responsible for her own trade; she is merely the supply of an inexorable demand to which the very agents of her persecution very likely contribute. She is alluded to by Homer and by Solomon and she was an ancient institution even in their time. We have no scheme for either her suppression or her salvation. We may shock the pious by confessing not so much horror as pity for this poor, bedraggled street sparrow seeking a precarious living from social excrement. Somehow, it does not seem quite fair, in addition to social ostracism, to make her the victim of police persecution of special severity, more particularly because no such system can eradicate the cause of which she is the effect. We rather incline to the policy of getting after the "man higher up." Certainly, if she is either to be reformed or controlled, it must be sympathetic assistance rather than oppression.

